# The role of Simpson grading in meningiomas after integration of the updated WHO classification and adjuvant radiotherapy

**DOI:** 10.1007/s10143-020-01428-7

**Published:** 2020-10-26

**Authors:** Felix Behling, Christina Fodi, Elgin Hoffmann, Mirjam Renovanz, Marco Skardelly, Ghazaleh Tabatabai, Jens Schittenhelm, Jürgen Honegger, Marcos Tatagiba

**Affiliations:** 1 Department of Neurosurgery, University Hospital Tuebingen, Eberhard-Karls-University Tuebingen, Hoppe-Seyler Street 3, Tuebingen, Germany; 2 Center for CNS Tumors, Comprehensive Cancer Center Tuebingen-Stuttgart, University Hospital Tuebingen, Eberhard-Karls-University Tuebingen, Tuebingen, Germany; 3 German Cancer Consortium (DKTK), DKFZ partner site Tuebingen, Tuebingen, Germany; 4 Department of Radiation Oncology, University Hospital Tuebingen, Eberhard-Karls-University Tuebingen, Tuebingen, Germany; 5 Interdisciplinary Division of Neuro-Oncology, University Hospital Tuebingen, Eberhard-Karls-University Tuebingen, Tuebingen, Germany; 6 Department of Neurology, University Hospital Tuebingen, Eberhard-Karls-University Tuebingen, Tuebingen, Germany; 7 Hertie Institute for Clinical Brain Research, Tuebingen, Germany; 8 Department of Neuropathology, University Hospital Tuebingen, Eberhard-Karls-University Tuebingen, Tuebingen, Germany

**Keywords:** Meningioma, Simpson grading, Prognosis, Progression-free survival, Recurrence, Radiotherapy

## Abstract

Since the introduction of the Simpson grading for the extent of resection in meningiomas in 1957, its usefulness in modern neurosurgery has been challenged. Especially, the updated WHO classification regarding brain invasion and the efficacy of radiation therapy has not been taken into account when evaluating the prognostic role of the Simpson grading in this era. We analyzed the clinical and histopathological data of 1571 meningiomas that were surgically resected in the authors’ institution between July 2003 and March 2017. Operative reports were reviewed regarding the extent of resection according to Simpson grading. Meningioma subtype according to the updated WHO classification of 2016 and clinical characteristics and time to tumor progression were analyzed. The mean follow-up was 38.4 months (range 1.2 to 195.6). A higher rate of tumor recurrence was observed for male gender, younger age, recurrent tumors, non-spinal tumor localization, higher WHO, and Simpson grades in the univariate analysis. In the multivariate analysis older age, recurrent tumors and higher WHO grades remained negative prognostic factors. Among the different Simpson grades, the relative risk for recurrence was highest for grade IV compared to all other grades (each *p* < 0.0001), while there was no difference between Simpson grades I and II. Adjuvant radiotherapy showed lower rates of tumor recurrence. Subtotal microsurgical resection remains an independent prognostic factor with a higher rate of tumor recurrence. The prognostic benefit of radical treatment of the dural attachment is questionable and needs to be considered when weighing the intraoperative risks of radicality.

**Supplementary Information:**

The online version contains supplementary material available at 10.1007/s10143-020-01428-7.

## Introduction

Meningioma remains the most common non-malignant primary tumor of the central nervous system, making up one-third of newly diagnosed tumors [11]. Complete tumor resection is the treatment of choice if surgically feasible [3]. However, depending on the location and infiltration into adjacent structures, a complete microsurgical excision is not always possible. Based on residual tumor or infiltrated dura a prognostic grading for the extent of resection was introduced by Simpson in 1957, which has been widely applied in neurosurgical practice since [16]. Several retrospective studies have assessed the prognostic role of the Simpson grading with differing results [1, 4, 10, 12]. It may seem obvious that leaving tumor tissue or infiltrated dura behind results in a significant risk of tumor regrowth. However, it remains uncertain where the prognostic role of Simpson grading stands today, especially in light of the established importance of adjuvant radiotherapy for selected cases [2, 3, 6, 13, 14] and the recent reclassification of former grade I meningiomas with brain invasion as grade II atypical meningiomas [7]. The aim of this single-center retrospective study was to analyze the prognostic impact of the extent of resection according to the Simpson grading in light of the updated WHO classification together with established prognostic factors and adjuvant radiotherapy.

## Materials and methods

We performed a retrospective analysis of all meningiomas that were surgically treated in the Department of Neurosurgery of the University Hospital Tübingen between July 2003 and March 2017. The study was approved by the Clinical Ethics Committee of the University of Tübingen (project number: 618/2014BO2). Gender, age, histopathological diagnosis, extent of resection (according to the Simpson grading system), tumor localization, time to radiographic tumor recurrence/progression, and adjuvant radiotherapy treatment between surgery and tumor recurrence were collected via an electronic patient data review. All samples underwent a neuropathological review according to the WHO classification of 2016. Statistical analysis was done with JMP® (Cary, NC: SAS Institute Inc., 1989) Statistical Discovery Software, version 14.2.0. The Pearson chi-squared and the log-rank test were used for univariate and the Wald test for multivariate analysis while a significance level of *α* < 0.05 was applied. A classification and regression tree (CART) analysis was used to define the optimal prognostic age cut off.

## Results

### Cohort characteristics

**Fig. 1:**
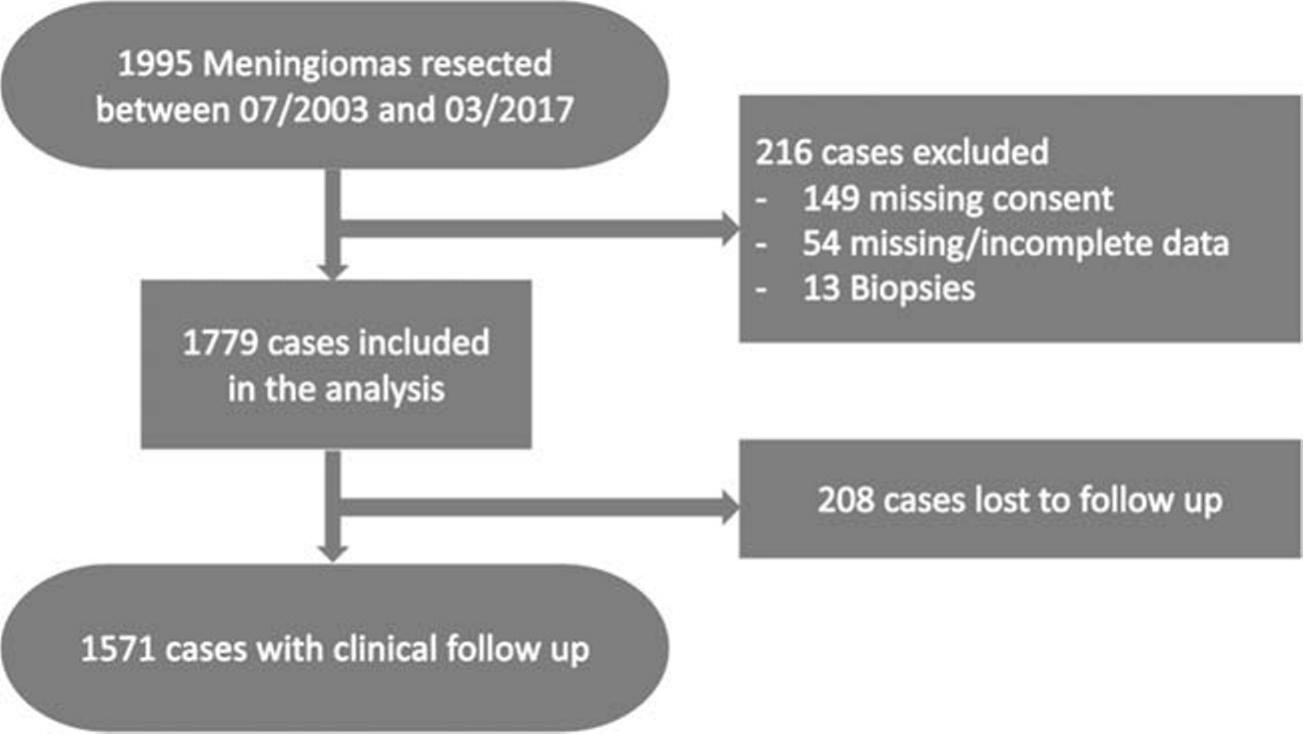
Consort diagram delineating the patients included in the study cohort

Overall, 1995 meningiomas were surgically resected in our department between July 2003 and March 2017. One hundred forty-nine patients without written consent for data analysis were excluded from the study. In 54 cases, the clinical data was incomplete or missing. Only 13 meningiomas were biopsied and the follow-up data was mostly incomplete or very short. Therefore, biopsied cases were also excluded completely. Two-hundred and eight cases were lost to follow-up. A consort diagram delineates the generation of the study cohort (Fig. 1).

A total of 1571 cases were eligible for further analysis and the characteristic of the cohort are displayed in Table 1. The mean follow-up was 38.4 months ranging from 1.1 to 195.6 months. The female to male ratio was 2.57 (1131/440) and the mean age 56.6 years ranging from 3.8 to 90.0 years. Recurrent meningiomas made up 13.4% (211/1360) of the patient cohort. Skull base meningiomas made up 51.9% (816/1571) followed by convexity/falx (38.7%, 608/1571) and spinal localization (9.4%, 147/1571). According to the WHO classification of 2016, 79.6% were grade I (1251/1571) while 18.8% were grade II and 1.6% grade III (295/1571 and 25/1571, respectively).

**Table 1:** Cohort characteristics and univariate analysis of tumor recurrence (chi-squared test)

	*N* (%)	Tumor recurrence *n* (%)		*p* value
		Yes	No	
Age				
≥ 45.26	1248 (79.4)	246 (19.7)	1002 (80.3)	< 0.0001*
< 45.26	323 (20.6)	104 (32.2)	219 (67.8)	
Gender				
Female	1131 (72.0)	201 (17.8)	930 (82.2)	< 0.0001*
Male	440 (28.0)	149 (33.9)	291 (66.1)	
Primary/recurrence				
Primary	1360 (86.6)	216 (15.9)	1144 (84.1)	< 0.0001*
Recurrence	211 (13.4)	134 (63.5)	77 (36.5)	
Tumor localization				
Skull base	816 (51.9)	191 (23.4)	625 (76.6)	< 0.0001*
Convexity/falx	608 (38.7)	151 (24.8)	457 (75.2)	
Spinal	147 (9.4)	8 (5.4)	139 (94.6)	
WHO classification 2016				
I	1251 (79.6)	187 (15.0)	1064 (85.0)	< 0.0001*
II	295 (18.8)	142 (48.1)	153 (51.9)	
III	25 (1.6)	21 (84.0)	4 (16.0)	
Adjuvant radiotherapy				
Yes	81 (5.2)	26 (32.1)	55 (67.9)	0.0283*
No	1489 (94.8)	323 (21.7)	1166 (78.3)	
Simpson grade				
I	376 (23.9)	61 (16.2)	315 (83.8)	< 0.0001*
II	408 (26.0)	34 (8.3)	374 (91.7)	
III	303 (19.3)	57 (18.8)	246 (81.2)	
IV	484 (30.8)	198 (40.9)	286 (59.1)	
V	0 (-)	0 (-)	0 (-)	

For *p*-values marked with an asterisk (*) the level of significance (α < 0.05) was reached

### Univariate analysis of tumor recurrence

The CART analysis revealed the age cut off at 45.26 years with the most pronounced difference regarding tumor recurrence. Younger patients showed a significantly higher risk of tumor recurrence in the univariate analysis (32.2% vs. 19.7%, *p* < 0.0001, Table 1). Male gender was associated with a higher risk of tumor recurrence as well (33.9% vs. 19.7%, *p* < 0.0001). Recurrent tumors showed an increased risk of tumor recurrence after the second resection compared to primary meningiomas after the first resection (63.5% vs. 15.9%, *p* < 0.0001). Spinal tumor location was associated with a lower risk of recurrence compared to the skull base and convexity/falx location (5.4% vs. 23.4% and 24.8%, respectively, *p* < 0.0001). Higher WHO grade was also associated with a higher probability of tumor recurrence (15.0%, 48.1%, and 84.0% for grade I, II, and III meningiomas, respectively, *p* < 0.0001). Cases that received adjuvant radiotherapy after microsurgical resection showed a significant negative prognostic impact in the univariate analysis (32.1% vs. 21.7%, *p* = 0.0283). These meningiomas were mostly of higher WHO grades (> 50%), were subtotally resected in 71.6%. Subtotal resections (Simpson grade IV) were associated with higher rates of tumor recurrence (40.9% for grade IV compared to 16.2%, 8.3%, and 18.8% for grades I, II, and III, respectively, *p* < 0.0001).

**Fig. 2:**
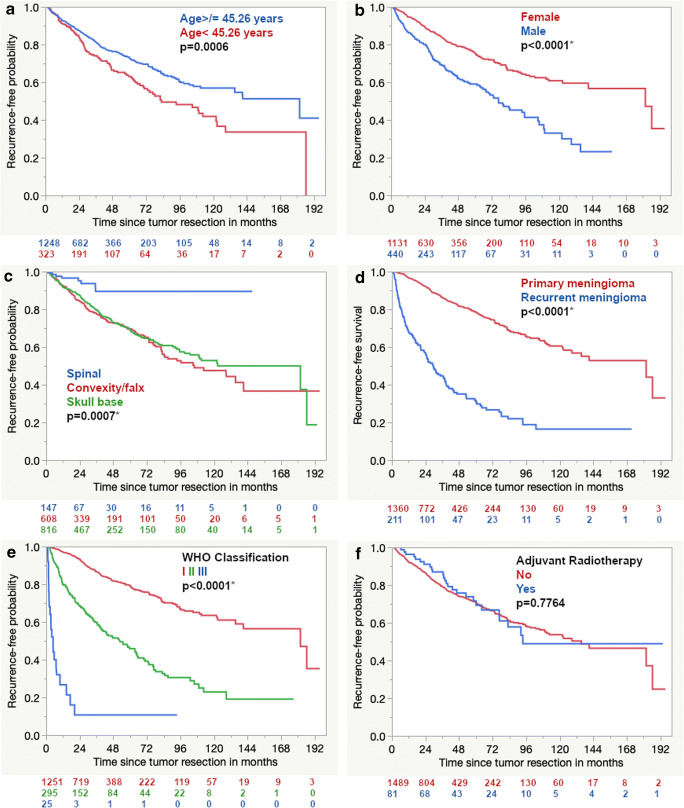
Kaplan-Meier curves for **a** age, **b** gender, **c** tumor localization, **d** primary/recurrent tumors, **e** WHO classification, and **f** adjuvant radiotherapy with corresponding *p* values of the log-rank test

Corresponding Kaplan-Meier analyses confirmed the results of the Pearson chi-squared test. Only the negative prognostic effect of adjuvant radiotherapy was not confirmed in the Kaplan-Meier curve (Fig. 2).

**Fig. 3:**
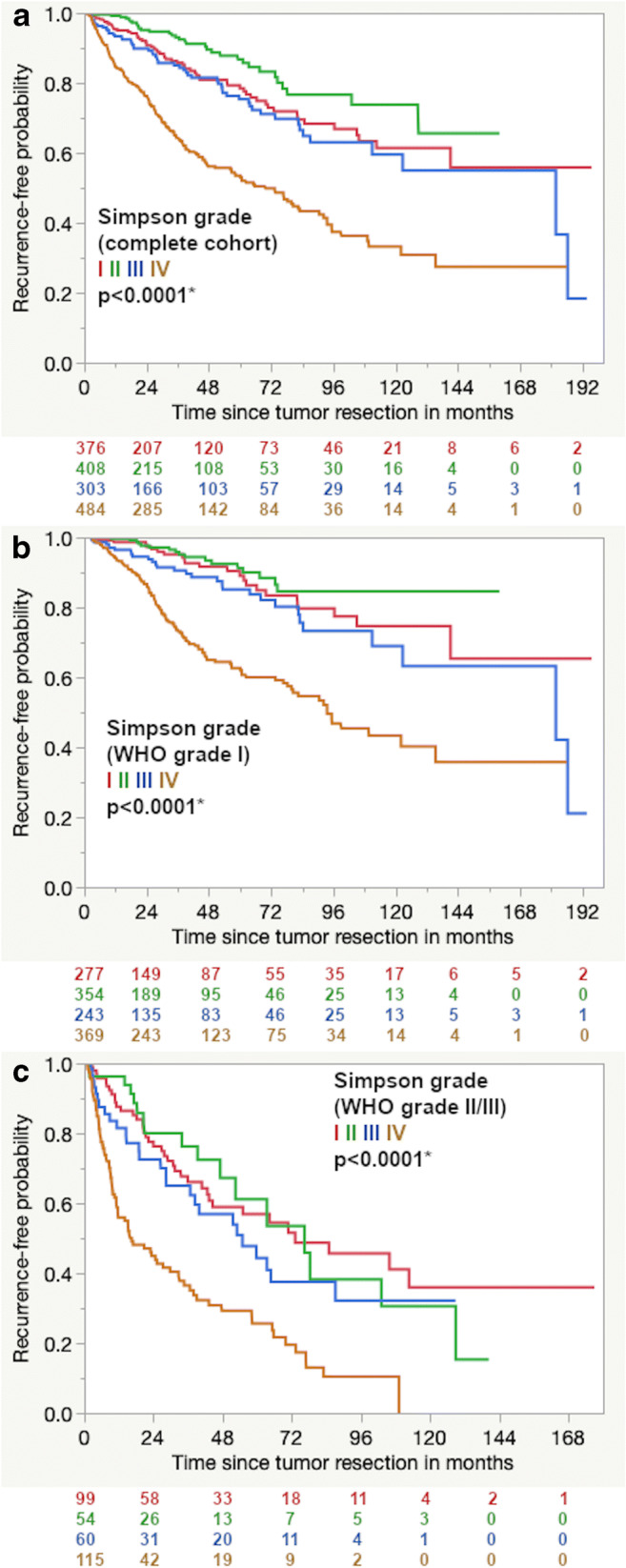
Kaplan-Meier curves for the extent of resection according to Simpson regarding the **a** complete cohort and subgroups adjusted to WHO grades **b** I and **c** II/III

The extent of resection according to Simpson in the Kaplan-Meier analysis shows that grade II resection has the best progression-free survival, even better than grade I (Fig. 3). It has to be emphasized that in this cohort over 80% of Simpson grade II resections are meningiomas of the skull base and the spine (Table 2). Both subgroups consist mostly of WHO grade I tumors (87.3% for skull base and 95.9% for spinal meningiomas) while 34.5% of meningiomas of the convexity and falx are tumors with a higher WHO grade (Supplementary Fig. 1). This has to be kept in mind for the univariate analysis. When adjusted for WHO grade (separate analysis for WHO grades I and II/III), the Kaplan-Meier curves show no difference in progression-free survival between Simpson grades I, II, and III (Fig. 3).

**Table 2:** Distribution of Simpson grades (chi-squared test)

	*N*	Simpson grade					*p* value (Prob > Chisq)
	1571 (100)	I	II	III	IV	V	
Age							
≥ 45.26 years	1248 (79.4)	295 (23.6)	339 (27.2)	247 (19.8)	367 (29.4)	0 (-)	0.0417*
< 45.26 years	323 (20.6)	81 (25.1)	69 (21.4)	56 (17.3)	117 (36.2)	0 (-)	
Gender							
Female	1131 (72.0)	291 (25.7)	312 (27.6)	209 (18.5)	319 (28.2)	0 (-)	0.0002*
Male	440 (28.0)	85 (19.3)	96 (21.8)	94 (21.4)	165 (37.5)	0 (-)	
Recurrent tumor							
Recurrence	211 (13.4)	43 (20.4)	18 (8.5)	26 (12.3)	124 (58.8)	0 (-)	< 0.0001*
Primary	1360 (86.6)	333 (24.5)	390 (28.7)	277 (20.4)	360 (26.5)	0 (-)	
Tumor location							
Skull base	816 (51.9)	142 (17.4)	239 (29.3)	119 (14.6)	316 (38.7)	0 (-)	< 0.0001*
Convexity/falx	608 (38.7)	234 (38.5)	99 (16.3)	133 (21.9)	142 (23.4)	0 (-)	
Spinal	147 (9.4)	0 (−)	70 (47.6)	51 (34.7)	26 (17.7)	0 (-)	
WHO							
I	1251 (79.6)	280 (22.4)	357 (28.5)	244 (19.5)	370 (29.6)	0 (-)	< 0.0001*
II	295 (18.8)	92 (31.2)	47 (15.9)	55 (18.6)	101 (34.2)	0 (-)	
III	25 (1.6)	4 (16.0)	4 (16.0)	4 (16.0)	13 (52.0)	0 (-)	
Adjuvant radiotherapy							
Yes	81 (5.2)	10 (12.4)	5 (6.2)	8 (9.9)	58 (71.6)	0 (-)	< 0.0001*
No	1489 (94.9)	365 (24.5)	403 (27.1)	295 (19.8)	426 (28.6)	0 (-)	

For *p*-values marked with an asterisk (*) the level of significance (α < 0.05) was reached

### Distribution of Simpson grade

The Simpson grade showed differing distribution between several subgroups (Table 2). A subtotal resection (Simpson grade IV) was more often reported in male patients (37.5 vs. 28.2, *p* = 0.0002) and in recurrent meningiomas (58.8 vs. 26.5, *p* < 0.0001). Additionally, a grade IV resection was more common for meningiomas located at the skull base compared to convexity/falx and spinal meningiomas (38.7 vs. 23.4 and 17.7, *p* < 0.0001). Adjuvant radiotherapy was mostly administered to patients where residual tumor tissue had to be left behind (71.6%) and also in selected cases of Simpson grades I, II, and III (12.4%, 6.2 and 9.9%, *p* < 0.0001) mostly because of higher WHO grade.

### Multivariate analysis of tumor recurrence

All factors were combined into a Cox proportional hazard model (Table 3). Male gender and younger age remained independent negative prognostic factors (*p* = 0.0107 and *p* = 0.0191, respectively). There was a significantly higher risk of tumor recurrence for skull base location compared to spinal meningiomas (*p* = 0.0475). Recurrent meningiomas are at an increased risk of another tumor recurrence when compared to primary meningiomas (*p* < 0.0001). Lower WHO grade as well as adjuvant radiotherapy were independent positive prognostic factors with a lower rate of tumor recurrence (each *p* < 0.0001).

**Table 3:** Multivariate analysis of tumor recurrence (Cox proportional hazard)

	Risk ratio (95%CI)	*p* value (Prob > Chisq)
Age < 45.26 years	1.32 (1.05–1.68)	0.0191*
Male gender	1.34 (1.07–1.68)	0.0107*
Recurrent tumor	3.66 (2.89–4.63)	< 0.0001*
Tumor location		
Skull base vs. spinal	2.06 (1.01–4.20)	0.0475*
Skull base vs. convexity/falx	1.18 (0.94–1.49)	0.1594
Convexity/falx vs. spinal	1.74 (0.84–3.60)	0.1345
WHO		
I vs. II	0.28 (0.22–0.36)	< 0.0001*
I vs. III	0.06 (0.04–0.11)	< 0.0001*
II vs. III	0.23 (0.14–0.37)	< 0.0001*
Adjuvant radiotherapy	0.31 (0.21–0.47)	< 0.0001*
Simpson grade		
II vs. I	0.82 (0.54–1.26)	0.3717
III vs. I	1.45 (1.00–2.10)	0.0490*
III vs. II	1.76 (1.15–2.70)	0.0095*
IV vs. I	2.92 (2.14–3.98)	< 0.0001*
IV vs. II	3.55 (2.45–5.15)	< 0.0001*
IV vs. III	2.01 (1.49–2.73)	< 0.0001*

For *p*-values marked with an asterisk (*) the level of significance (α < 0.05) was reached

Subtotal tumor resection (Simpson grade IV) was associated with 2–3 times higher risk of tumor recurrence when compared to grades I through III (each *p* < 0.0001). There was no significant difference between grades I and II, while the risk of tumor recurrence was 1.4-fold between grades III and I and 1.8-fold between grades III and II (*p* = 0.0490 and *p* = 0.0095, respectively.

## Discussion

The grading of the extent of resection for meningiomas has been used by neurosurgeons for over 60 years [16]. Since its implementation in 1957, the knowledge on prognosis and risk of tumor recurrence has evolved. First of all, the WHO classification has been updated for meningiomas in 2016 [7]. Furthermore, radiation therapy has become an established primary treatment option for selected cases and an efficacious adjunct after subtotal resection of more aggressive tumors [3]. Both developments need to be considered when analyzing prognostication in meningioma.

Several studies have assessed the role of the Simpson grading and produced differing results. Nanda et al. showed that a radical resection according to Simpson grade I in WHO grade I meningiomas is still of prognostic significance when compared to all other Simpson grades combined [10]. Although it seems reasonable to resect or coagulate the complete dural attachment for a more radical resection (Simpson grade I or II, respectively), there is also growing evidence that it is of no benefit regarding tumor recurrence. A retrospective analysis of 248 grade I meningiomas revealed that there was no prognostic difference in tumor recurrence between Simpson grades I, II, and III, while only grade IV was associated with a significantly shorter progression-free survival [12]. In contrast to these findings, a retrospective analysis of 900 meningiomas by Gousias et al. emphasized the prognostic effect of a radical resection according to Simpson grade I vs. II and also noted only a small difference between grades III and IV. It has to be emphasized that the authors produced data of a more complete cohort including all WHO grades and long follow-up intervals compared to the before-mentioned retrospective studies. However, the study is based on the old WHO classification and therefore did not take into account the prognostic effect of brain invasion and furthermore, the role of adjuvant radiotherapy [4].

The univariate analysis suggested the best recurrence-free survival for tumor resections according to Simpson grade II, even better than Simpson grade I. However, the majority of our cohort consists of skull base meningiomas that are usually not radically resected (not Simpson grade I) and are more likely WHO grade I than tumors of the convexity and the falx. After adjusting for the differences in WHO grade distribution, there was no difference in recurrence-free survival between Simpson grades I, II, and III in the Kaplan-Meier analysis.

Our data showed the most pronounced prognostic effect between Simpson grade IV and all other grades. In the multivariate analysis, there was no prognostic effect of dural resection (I) compared to coagulation (II), while dural coagulation (II) had a benefit compared to leaving the dural attachment untreated (III). These findings suggest that proper coagulation of the dural attachment seems to be still quite effective and may be equivalent to radical resection of the dural attachment. However, there is obviously a wide variety of technical approaches to dural coagulation. Specific standards regarding intensity and duration for optimal thermal injury necessary for efficacious recurrence control do not exist. The technique of coagulation of the dura is usually based on each neurosurgeon’s experience and the proximity to critical structures. Furthermore, the extent of dural infiltration can usually not be differentiated from reactive dural changes intraoperatively, unless extensive sampling and frozen section evaluations are performed, which is usually not done. The development of intraoperative tools to assess the dural infiltration with a high sensitivity would be of great benefit to apply the radical treatment of the dura more accurately. The use of intraoperative 5-ALA guidance for resection of residual meningioma tissue and bone invasion has been described [15]. Although the reliability and role of 5-ALA fluorescence in meningioma has been reviewed quite critically recently [9], trials of larger cohorts have produced more convincing results, also concerning the detection of dural infiltration [17].

It is natural for neurooncological surgeons to have the urge for radical resection if technically feasible. But the risk for the patient can be increased as demonstrated in a meta-analysis covering 896 spinal meningiomas. Barber et al. showed that radical resection of spinal meningiomas according to Simpson grade I was associated with a higher rate of complications while no prognostic advantage was shown compared to grade II [1]. Since spinal meningiomas recur to a lesser extent and are in ultimate proximity to highly functional neural tissue, the results are not fully comparable to intracranial meningiomas [8]. However, Barber et al. raise awareness that radicality can come with a price and the benefit may be questionable [1]. Our data underline the necessity to be radical especially not to leave macroscopic tumor tissue behind (highest relative risk of progression for Simpson grade IV compared to all other grades). The high risk of another tumor recurrence for recurrent meningiomas is also demonstrated in our results and confirms the findings of a large retrospective analysis recently published by Lemee and colleagues that showed the need to make use of the possibility of radical resection during the first attempt [5].

There are two novel aspects of our retrospective analysis. To our knowledge, the prognostic impact of the Simpson grading has not yet been evaluated in a comprehensive cohort with the integration of the updated WHO classification of 2016. Additionally, the integration of the prognostic effect of adjuvant radiotherapy is also a novel and important aspect, since its clinical efficacy has been established for the control of residual or higher grade meningiomas [2, 3]. Our data indicate a protective effect for adjuvant radiotherapy in the multivariate analysis after integration of all other prognostic factors. The negative prognostic effect in the univariate analysis is explained by the high proportion of higher grade or subtotally resected meningiomas in this subgroup and is reversed in the multivariate analysis when all confounders are taken into account. Our data do not support a generalization of the prognostic effect of adjuvant radiotherapy but the efficacy in selected cases is demonstrated.

Most importantly, after the integration of all prognostic variables including adjuvant radiation therapy and the updated WHO classification, the strong prognostic effect of complete tumor resection (< Simpson grade IV) remains. This is a clear confirmation that a complete resection of tumor tissue is important to achieve the best possible result and prognosis for our patients.

### Limitations and strengths

The main limitation of this study is its retrospective nature. Due to our center’s clinical focus on skull base tumors, meningiomas in this location, and especially recurrent tumors are overrepresented. The single-center design is therefore a limitation of this study. On the other hand, as a single-center analysis in a high-volume institution, clinical management, follow-up, and handling of data were homogeneous. Furthermore, our cohort has similar properties when compared to established clinical characteristics (age, gender, WHO grade). Only 208 cases were lost to follow-up. The clinical characteristics of this subgroup are displayed in the Supplementary Table 1. A follow-up of 5 years or longer was only reached for 650 cases. It is likely that especially patients with recurring and difficult tumors were followed more thoroughly and therefore had a longer follow-up, while more favorable clinical courses were managed without further contact with our center. However, in the univariate analysis of the complete cohort and the 5-year follow-up cohort, the significance of prognostic factors was the same. The strengths of the study are its high case number and inclusion of the WHO classification of 2016 and prognostic effect of adjuvant radiotherapy.

## Conclusion

Incomplete resection of meningiomas remains an independent prognostic factor. The prognostic benefit of radical treatment of the dural attachment is questionable and needs to be weighed against the intraoperative risk for each case. Selected adjuvant radiotherapy is an independent positive prognostic factor for recurrence-free survival if applied for selected cases.

## Electronic supplementary material

Supplementary Fig. 1 Distribution of WHO grade varies significantly between different meningiomas localizations (PNG 22 kb).

ESM 1 (DOC 33 kb).

## Data Availability

The dataset is available upon request.
