# Supplementary figures and images for: The role of Simpson grading in meningiomas after integration of the updated WHO classification and adjuvant radiotherapy
Source: Neurosurg Rev. 2020 Oct 26;44(4):2329–36. doi: 10.1007/s10143-020-01428-7 (PMC8338836; doi:10.1007/s10143-020-01428-7)

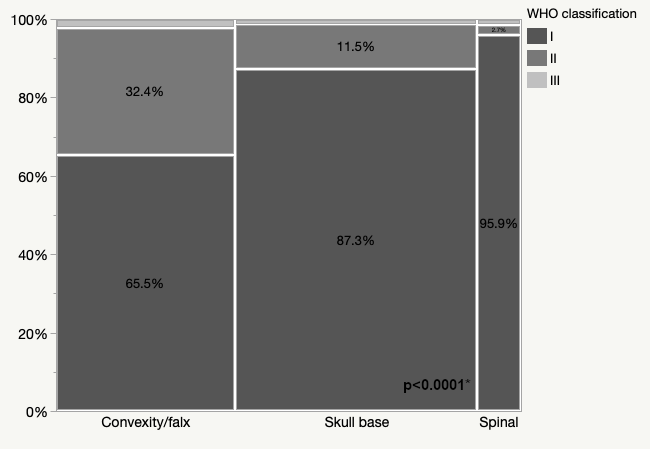

Supplement: Supplementary file 1 — Distribution of WHO grade varies significantly between different meningiomas localizations (PNG 22 kb). [file 10143_2020_1428_MOESM1_ESM.png]
