# Supplementary material for: The role of Simpson grading in meningiomas after integration of the updated WHO classification and adjuvant radiotherapy
Source: Neurosurg Rev. 2020 Oct 26;44(4):2329–36. doi: 10.1007/s10143-020-01428-7 (PMC8338836; doi:10.1007/s10143-020-01428-7)
Supplement: Supplementary file 2 — (DOC 33 kb). [file 10143_2020_1428_MOESM2_ESM.doc]

Supplementary Table 1: Excluded cases that were lost to follow up compared to the study cohort.

|  | **Lost to follow up** | **Study cohort** |
| --- | --- | --- |
|  | **N (%)** | **N (%)** |
| **Age**  >= 45.26  < 45.26  **Gender**  Female  Male    **Primary/Recurrence**  Primary  Recurrence  **Tumor localization**  Skull base  Convexity/falx  Spinal  **WHO classification 2016**  I  II  III  **Simpson grade**  I  II  III  IV  V | 168 (80.8)  40 (19.2)  138 (66.3)  70 (33.7)  185 (88.9)  23 (11.1)  101 (48.6)  70 (33.6)  37 (17.8)  162 (77.9)  44 (21.2)  2 (1.0)  49 (23.6)  50 (24.0)  51 (24.5)  58 (27.9)  0 (-) | 1248 (79.4)  323 (20.6)  1131 (72.0)  440 (28.0)  1360 (86.6)  211 (13.4)  816 (51.9)  608 (38.7)  147 (9.4)  1251 (79.6)  295 (18.8)  25 (1.6)  376 (23.9)  408 (26.0)  303 (19.3)  484 (30.8)  0 (-) |
